# Allogeneic Periodontal Ligament Stem Cell Therapy for Periodontitis in Swine

**DOI:** 10.1002/stem.512

**Published:** 2010-08-31

**Authors:** Gang Ding, Yi Liu, Wei Wang, Fulan Wei, Dayong Liu, Zhipeng Fan, Yunqing An, Chunmei Zhang, Songlin Wang

**Affiliations:** a Molecular Laboratory for Gene Therapy and Tooth Regeneration, Capital Medical University School of StomatologyBeijing, People's Republic of China; b Department of Immunology andCapital Medical University School of Basic Medical SciencesBeijing, People's Republic of China; c Biochemistry and Molecular Biology, Capital Medical University School of Basic Medical SciencesBeijing, People's Republic of China

**Keywords:** Periodontal ligament stem cells, Immunogenicity, Immunosuppression, Tissue engineering

## Abstract

Periodontitis is one of the most widespread infectious diseases in humans. It is the main cause of tooth loss and associated with a number of systemic diseases. Until now, there is no appropriate method for functional periodontal tissue regeneration. Here, we establish a novel approach of using allogeneic periodontal ligament stem cells (PDLSCs) sheet to curing periodontitis in a miniature pig periodontitis model. Significant periodontal tissue regeneration was achieved in both the autologous and the allogeneic PDLSCs transplantation group at 12 weeks post-PDLSCs transplantation. Based on clinical assessments, computed tomography (CT) scanning, and histological examination, there was no marked difference between the autologous and allogeneic PDLSCs transplantation groups. In addition, lack of immunological rejections in the animals that received the allogeneic PDLSCs transplantation was observed. Interestingly, we found that human PDLSCs fail to express human leukocyte antigen (HLA)-II DR and costimulatory molecules. PDLSCs were not able to elicit T-cell proliferation and inhibit T-cell proliferation when stimulated with mismatched major histocompatibility complex molecules. Furthermore, we found that prostaglandin E2 (PGE2) plays a crucial role in PDLSCs-mediated immunomodulation and periodontal tissue regeneration in vitro and in vivo. Our study demonstrated that PDLSCs possess low immunogenicity and marked immunosuppression via PGE2-induced T-cell anergy. We developed a standard technological procedure of using allogeneic PDLSCs to cure periodontitis in swine. Stem Cells 2010;28:1829–1838

## INTRODUCTION

Periodontitis is one of the most widespread infectious diseases in humans. It is the main cause of tooth loss and is associated with a number of systemic diseases such as diabetes and cardiovascular disease [1]. Thus far, no appropriate method has been developed to provide functional and predictable periodontal tissue regeneration. Several regenerative approaches, including guided tissue regeneration, application of enamel matrix derivative, and various growth factors, were proposed to treat periodontal disease and acquired favorable results in clinical trials and animal models [2–5]. Recently, the identification of periodontal ligament stem cells (PDLSCs) and other tooth-related stem cells has provided new prospect and appropriate cell sources for periodontal tissue regeneration [6–8]. Previously, we generated periodontitis in swine model, and we have used autologous PDLSCs to cure periodontitis with significant periodontal tissue regeneration in swine [9,10]. However, sources of autologous PDLSCs are limited, particularly for aged patients, largely impeding the clinical application of this approach. Thus, it is critical to develop a feasible allogeneic cell-based method for the treatment of periodontitis.

In the present study, we have generated a periodontitis model and transplanted cell sheets derived from allogeneic PDLSCs for the treatment of periodontitis in miniature pigs. In addition, we have assessed immunogenicity and immunomodulation of human PDLSCs (hPDLSCs) and miniature pig PDLSCs (pPDLSCs) and investigated their contribution to PDLSC-mediated therapies. Our data demonstrate that allogeneic pPDLSC sheets are able to provide appropriate therapy for periodontitis with significant periodontal tissue regeneration and low immunogenicity. We have developed a standard technological procedure for the potential application of allogeneic PDLSCs in the treatment of periodontitis-induced bone defects.

## MATERIALS AND METHODS

### Antibodies

Antibodies used in this study are described in the Supporting Information Materials and Methods.

### PDLSC-Mediated Treatment for Swine Periodontitis

This study was reviewed and approved by the Animal Care and Use Committee of Capital Medical University. Female Wuzhishan and male Guizhou inbred miniature pigs (minipig; 6- to 8-months old, weighing 30–40 kg) were obtained from the Institute of Animal Science of the Chinese Agriculture University. Minipigs were kept under conventional conditions with free access to water and a regular supply of soft food. Minipig PDLSCs (pPDLSCs) were isolated from canine and cultured as described previously [9]. We seeded 1.0 × 10^6^ pPDLSCs onto 100-mm culture dishes. On confluency, 40 mg of hydroxyapatite/tricalcium phosphate (HA/TCP; Biomedical Materials and Engineering Center of Wuhan University of Technology, Wuhan, China, http://www.whut.edu.cn) were added, and the cells were cultured for several days to obtain the cell sheets naturally formed when pPDLSCs proliferate in the presence of HA/TCP. Two cell sheets were used to repair one defective area. We generated periodontitis lesions in 15 female Wuzhishan inbred minipigs as previously reported [9], for a total of 30 defects. These defects were randomly assigned to five groups: (a) control group (six defects in three minipigs) with initial periodontal therapy only; (b) HA/TCP group (six defects in three minipigs), in which the defects were treated with flap surgery, transplantation of HA/TCP scaffolds, and covering of the defects with gelatin membranes (Nangjing Jingling Medical Co., Nanjing, China, http://www.jlpharm.com); (c) HA/TCP scaffolds + autologous pPDLSCs group (six defects in three minipigs), in which the defects were treated with flap surgery, transplantation of autologous pPDLSC sheets combined with HA/TCP scaffolds, and covering of the defects with gelatin membranes; (d) HA/TCP scaffolds + allogeneic Guizhou minipig pPDLSCs group (six defects in three minipigs), in which the defects were treated with flap surgery, transplantation of an allogeneic male Guizhou minipig pPDLSC sheet combined with HA/TCP scaffolds, and covering of the defects with gelatin membranes; (e) HA/TCP scaffolds + autologous heterogenic minipig periodontal ligament cells (pPDLCs) group (six defects in three minipigs), in which the defects were treated with flap surgery, transplantation of autologous pPDLCs sheets combined with HA/TCP scaffolds, and covering of the defects with gelatin membranes. A schematic illustration of the time line of the procedures conducted for this study is presented in Supporting Information Figure 1.

### Clinical Assessment of Periodontal Tissue Regeneration

Clinical assessments, including probing depth, gingival recession, and attachment loss, were measured on all experimental teeth prior to the generation of the periodontitis models (week −4), prior to the treatment (week 0), and 12 weeks post-transplantation. Routine blood tests (white blood cells, red blood cells, platelets, and the concentration of hemoglobin), routine biochemical tests (aspartate aminotransferase, alanine aminotransferase, total protein, blood urea nitrogen, and creatinine), immunoglobulin (IgE, IgG, IgA, IgM), and T cell-related markers (percentage of CD3^+^ cells, CD4^+^ cells, CD8^+^ cells, and the expression of the activated T-cell marker CD40L) were examined at week −4 and at 1–7 days, 2 weeks, 4 weeks, 8 weeks, and 12 weeks post-transplantation.

### Imaging and Histological Assessments

Bone regeneration was examined by computed tomography (CT; Siemens, Germany, http://www.medical.siemens.com) scanning upon transplantation (week 0) as well as 12 weeks post-transplantation. At 12 weeks post-transplantation, all animals were sacrificed; samples from the experimental areas were harvested, fixed with 4% formaldehyde, decalcified with 10% EDTA (pH 8.0), and embedded in paraffin. Sections (5 μm) were deparaffinized and stained with H&E. For quantification of bone formation, the extent of bone within each section was analyzed semiquantitatively by histomorphometric techniques as described previously [11]. Ten representative areas at ×5 magnification in each group were used. The area of bone formation was expressed as the percentage of bone in the periodontium in the sections.

### Cell Tracing in Regenerated Periodontal Tissues

Details of the immunofluorescent assays were described in Supporting Information Materials and Methods.

### Quantitative Reverse Transcription Polymerase Chain Reaction

Quantitative reverse transcription polymerase chain reaction (RT-PCR) assays for hepatocyte growth factor, transforming growth factor β1, interleukin (IL)-10, and cyclooxygenase 2 (COX-2) are described in the Supporting Information Materials and Methods.

### Human PDLSCs and Heterogenic PDLCs

All protocols for the handling of human tissue were approved by the Research Ethical Committee of Capital Medical University, China. Isolation and culture of human PDLSCs [6] and heterogenic periodontal ligament cells (hPDLCs) are performed as previous report [12]. All cells used in this study were at 3–4 passages. For each experiment, the same passages hPDLSCs and hPDLSc were used.

### Peripheral Blood Mononuclear Cells

Peripheral blood mononuclear cells (PBMCs) from healthy donors were obtained by gradient centrifugation separation method. In brief, 10 ml fresh heparinized peripheral blood was diluted with an equal volume of phosphate buffered saline (PBS). Five milliliters of diluted blood was carefully layered on 5 ml Ficoll (1.077 g/ml; Dingguo, Beijing, China, http://www.dingguo.com) for centrifugation at 900*g* for 30 minutes. The PBMCs layer was separated and washed with five volumes of PBS for three times, and precipitated cells were resuspended in Roswell Park Memorial Institute (RPMI)-1640 medium (GIBCO, Carlsbad, CA, http://www.invitrogen.com) containing 10% FBS, 20 mol/l HEPES, 2 mmol/l glutamine, 100 U/ml penicillin, and 100 μg/ml streptomycin (Invitrogen).

### Flow Cytometry Analysis of Cell Surface Markers

To characterize the expression profiles of surface molecules, hPDLSCs were harvested, and cell aliquots (1.0 × 10^6^ cells) were incubated with monoclonal antibodies against HLA-I, HLA-II DR, CD80, CD86, STRO-1, CD90, or CD146 for 1 hour at room temperature. After washing with PBS, the cells were incubated with fluorescein isothiocyanate-conjugated goat anti-mouse IgG, M, A antibodies for 30 minutes in the dark at room temperature. Antibodies were used in the concentrations suggested by the manufacturers. The expression profiles were analyzed by fluorescein-activated cell sorter Calibur flow cytometry (BD Inmmunocytometry Systems, San Jose, CA, http://www.bd.com).

### Multipotent Differentiation

Multilineage differentiation assays toward osteogenic and adipogenic pathways were performed as previously reported [10]. To detect osteogenic differentiation, calcification of the extracellular matrix was checked via von Kossa staining. Oil red O staining was used to identify lipid-laden fat cells.

### Immune Assays

5.0 × 10^4^ hPDLSCs and hPDLCs were irradiated (20 Gy; Varian, Palo Alto, CA, http://www.varianinc.com) before being cultured with allogeneic T cells. Then, hPDLSCs/hPDLCs and an equal number of PBMCs were cocultured in triplicate in a 96-well U-bottomed plate for 5 days in 0.2 ml RPMI-1640 (GIBCO, Carlsbad, CA, http://www.invitrogen.com). The plates were pulsed with 1 μCi/well ^3^H-thymidine (^3^H-TdR; Chinese Institute of Atomic Energy, Beijing, China, http://www.ciae.ac.cn) 18 hours before harvesting. Cells were harvested over glass fiber filters, and ^3^H-TdR incorporation was measured using a liquid scintillation counter (Wallsc, PerkinElmer, Wellelsy, MA, http://www.perkinelmer.com). Results of ^3^H-TdR incorporation are presented as mean counts per minute ± SD.

A mitogen proliferative assay was used to assess the effect of hPDLSCs/hPDLCs on T-cell proliferation. PBMCs (5.0 × 10^4^) stimulated by 0.5 μg/ml phytohemagglutinin (PHA; Sigma-Aldrich, St Louis, MI, http://www.sigma-aldrich.com) were mixed at various stimulator-responder ratios with autologous hPDLSCs/hPDLCs; 1.0 × 10^4^, 5.0 × 10^4^, 2.5 × 10^5^, and 5.0 × 10^5^ hPDLSCs/hPDLCs were added. A total of 1 μCi ^3^H-TdR was added into each well 18 hours prior to harvesting. The cells were harvested on day 5 and ^3^H-TdR incorporation was measured as described earlier.

To evaluate delayed addition of hPDLSCs/hPDLCs affected T-cell proliferation, hPDLSCs/hPDLCs (5.0 × 10^4^) were added in a 1:1 ratio to 2-day-old cultures of PBMCs stimulated by 0.5 μg/ml PHA. Prior to the last 18 hours of three additional culture days, 1 μCi ^3^H-TdR was added to the wells, followed by cell harvesting and measurement of ^3^H-TdR incorporation.

To study the effects of hPDLSCs/hPDLCs on a two-way mixed lymphocyte reaction (MLR), hPDLSCs/hPDLCs from the third person (third-party) were added at the beginning of the experiments in a final volume of 0.2 ml RPMI-1640. PBMCs (5.0 × 10^4^) from two individuals were incubated with an equal number of hPDLSCs/hPDLCs from third party. The proliferation of responder cells was assessed after 5 days; the cells were pulsed during the last 18 hours with ^3^H-TdR (1 μCi/well). The harvesting of cells and the measurement of ^3^H-TdR incorporation were carried out as described earlier.

### Restimulation of T cells Following Culture with hPDLSCs

To test whether the hPDLSC-induced effect on T-cell proliferation was reversible, PBMCs (5.0 × 10^4^) were initially incubated with an equal number of hPDLSCs and 0.5 μg/ml PHA for 5 days. T cells were harvested and cultured with PHA (0.5 μg/ml) or recombinant human IL-2 (50 U/ml; R&D systems, Minneapolis, MN, http://www.rndsystems.com). After 2 days, the cells were pulsed with ^3^H-TdR for further 18 hours, followed by measurement of ^3^H-TdR incorporation.

### Transwell Cultures

Transwell chambers with a 0.4-μm pore size membrane (Costar, Cambridge, MA, http://www.corning.com) were used to physically separate the PBMCs from the hPDLSCs. PBMCs (5.0 × 10^4^) were seeded with PHA (0.5 μg/ml) in the upper chamber, and hPDLSCs (5.0 × 10^4^) were placed in the bottom chamber. After 5 days of coculture, T cells were harvested, placed in 96-well plates, and pulsed with ^3^H-TdR for further 18 hours to measure the ^3^H-TdR incorporation.

### Measurement of Soluble Factors and Neutralization Assay

Enzyme-linked immunosorbent assays (ELISAs) were used to quantify HGF, TGF-β1, prostaglandin E2 (PGE2), and IL-10. Then, neutralizing monoclonal antibodies or inhibitors against specific soluble factors were added to a coculture containing PBMCs (5.0 × 10^4^), hPDLSCs (5.0 × 10^4^), and PHA (0.5 μg/ml) in Transwell chambers. Experimental details were described in the Supporting Information Materials and Methods.

### Foxp3+ T-Cell Measurement

Flow cytometry was used to determine the percentage of Foxp3+ T cells at 5 days post coculture with PBMCs (5.0 × 10^4^) and PHA (0.5 μg/ml) or coculture with PBMCs (5.0 × 10^4^), PHA (0.5 μg/ml), and allogeneic hPDLSCs (5.0 × 10^4^).

### Determination of the Percentage of Apoptotic T Cells

The percentage of apoptotic T cells at 5 days postculture with hPDLSCs and PHA was evaluated using the Annexin V-Fluos staining kit (Roche Diagnostics, Penzberg, Germany, http://www.roche-applied-science.com) according to the manufacturer's instructions.

### Statistical Analysis

Statistical significance was assessed by two-tailed Student's *t*-test or analysis of variance; a *p* value less than .05 was considered statistically significant.

## RESULTS

### Allogeneic PDLSC-Mediated Therapy for Periodontitis

**Figure 1:**
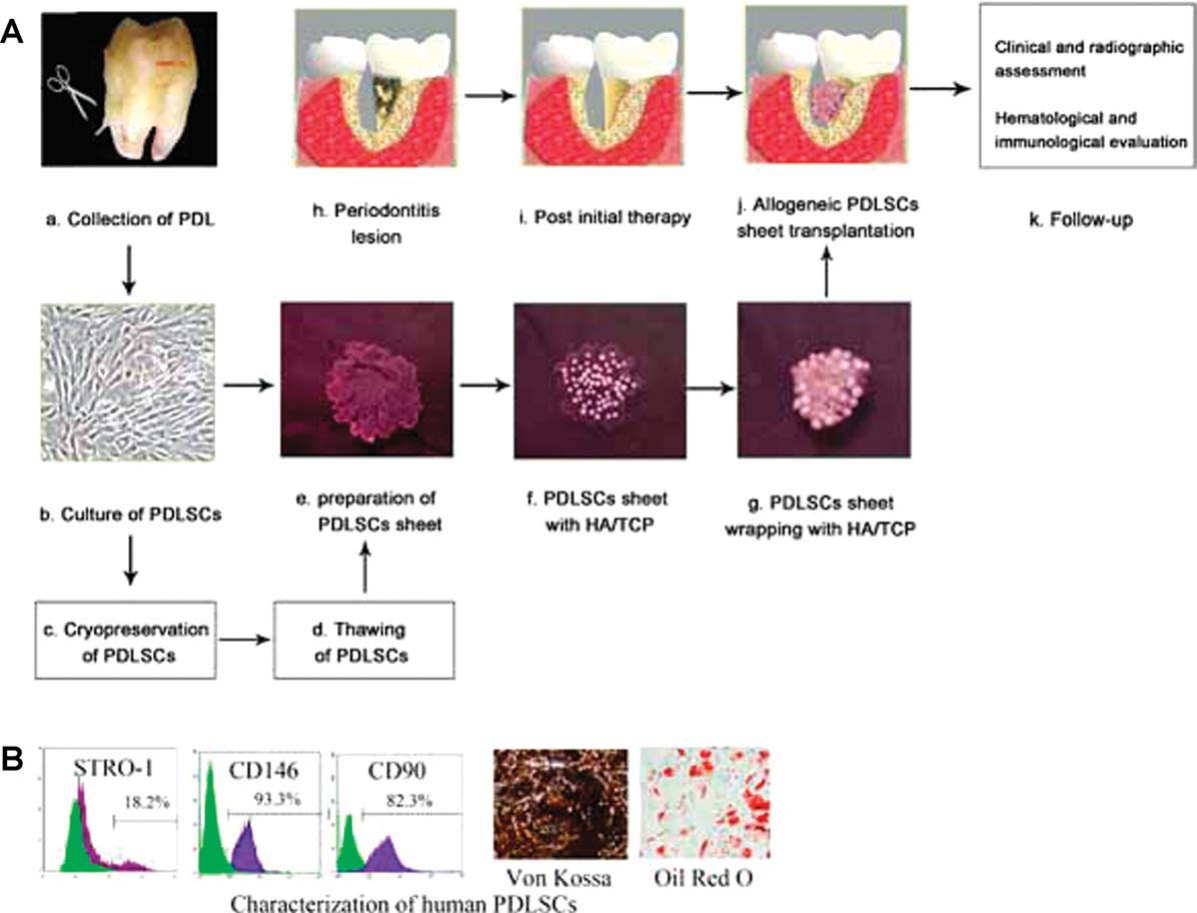
A standard procedure for the application of allogeneic PDLSCs in the treatment of periodontitis and characterization of PDLSCs. **(A):** (a) Collection of PDL. Normal impacted third molars were collected from patients aged 18–28 years. PDL was gently separated from the root surface. (b) Culture of PDLSCs. PDLSCs were isolated as previously reported. Fifteen days after primary culture, the number of PDLSCs from one impacted third molar was approximately 4.90 ± 0.34 × 10^5^ (P0; *n* = 10). Following another 15-day culture, the number of PDLSCs (P3) increased to 8.86 ± 0.46 × 10^6^ (*n* = 10). (c) Cryopreservation of PDLSCs. Third-passage PDLSCs were cryopreserved with 10% DMSO and 90% FBS and stored in liquid nitrogen. (d) PDLSCs thawing. After thawing, PDLSCs were examined for mycoplasma, bacteria, colony-forming efficiency, profiles of mesenchymal stem cell markers, and karyotype. (e) Preparation of PDLSC sheet. Three different cell amounts (1 × 10^5^, 1 × 10^6^, and 2 × 10^6^, *n* = 3) were cultured for 12–15 days in 100-mm culture dishes; cell sheets formed in the 1 × 10^6^ and 2 × 10^6^ groups but not in the 1 × 10^5^ group. Therefore, 1 × 10^6^ PDLSCs were seeded into 100-mm culture dishes for 15 days. (f) Forty milligrams of HA/TCP was added to the cultures. (g) The whole view of the PDLSC sheet with HA/TCP. (h) Buccal view of periodontitis lesion. After initial periodontal therapy (i), two allogeneic PDLSC sheets with HA/TCP were transplanted to a periodontal lesion sized 3 mm × 5 mm × 7 mm (j). (k) Follow-up included clinical and radiographic assessments and hematological and immunological evaluations. **(B):** PDLSCs were positive for STRO-1, CD146, CD90 and could advance into osteogenic and adipogenic differentiation under inductive medium. Abbreviations: HA/TCP, hydroxyapatite/tricalcium phosphate; PDL, periodontal ligament; PDLSC, periodontal ligament stem cell.

We developed a cell-based procedure for using allogeneic PDLSCs to treat periodontitis (Fig. 1A). PDLSCs were isolated from minipig canines or normal human impacted third molars and cultured to the third passage for use. The number of hPDLSCs and pPDLSCs from one tooth (P3) was 8.86 ± 0.46 × 10^6^ (*n* = 10) and 8.23 ± 0.68 × 10^6^ (*n* = 10), respectively. In this study, we utilized flow cytometry to indicate that PDLSCs were positive for STRO-1, CD146, which only expressed in early progenitors of culture expanded mesenchymal stem cells [6–8,10,13,14]. In addition, hPDLSCs expressed positive mesenchymal stem cell-associated markers CD90 [15]. Osteogenic differentiation of hPDLSCs was detected by nodular structures formation stained black via von Kossa staining, indicating calcium accumulation in vitro. PDLSCs were also found to possess the potential to develop into Oil red O-positive lipid-laden fat cells following 4 weeks of culture with an adipogenic-inductive medium (Fig. 1B). Cryopreservation did not affect the biological and immunological properties of the PDLSCs (data not shown), the same as human apical papilla stem cells [16]. To prepare the PDLSC sheet, 1 × 10^6^ PDLSCs were cultured in a 100-mm culture dish for 15 days, and then 40 mg HA/TCP particles were added to the dishes to increase plasticity of the cell-scaffold mixture (Fig. 1Ae, 1Af, 1Ag). After a surgical procedure to remove infectious periodontal tissues (Fig. 1Ah, 1Ai), allogeneic PDLSC sheet (with intact cell-cell junctions and deposited extracellular matrix) was applied to the periodontal defective areas for tissue regeneration (Fig. 1Aj).

**Figure 2:**
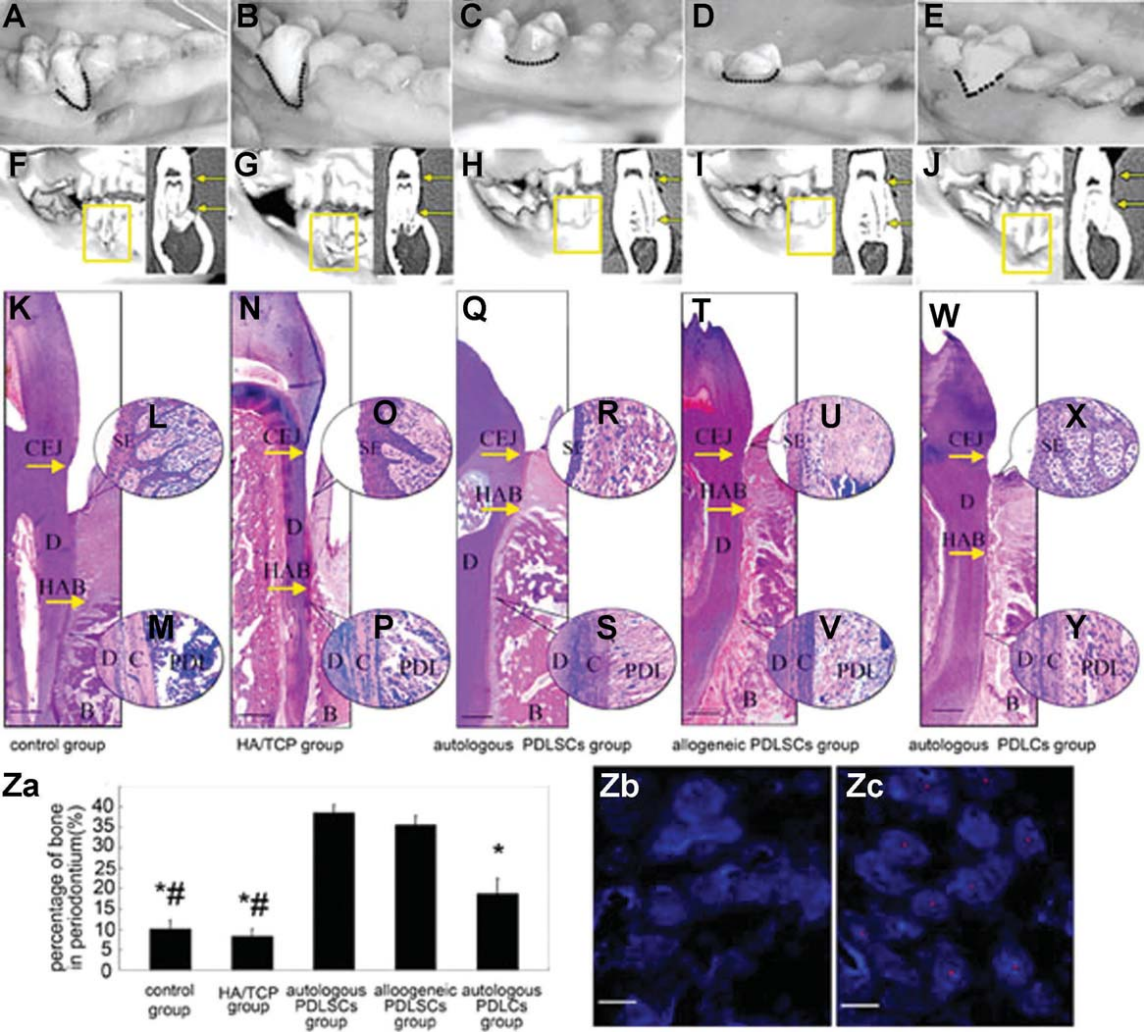
Regeneration of periodontitis defects mediated by allogeneic miniature pig PDLSCs (pPDLSCs). **(A–E):** Intraoral photographs indicated that 12 weeks after transplantation, autologous **(C)** or allogeneic **(D)** pPDLSC-mediated periodontal tissue regeneration was close to normal level. Only limited periodontal tissues were regenerated in the control group **(A)**, HA/TCP group **(B)**, and partially regenerated periodontal tissues in pPDLCs group **(E)**. CT images showed that limited bone formation was found in the control group **(F)** and the HA/TCP group **(G)** 12 weeks after pPDLSC transplantation. Autologous pPDLSCs **(H)** or allogeneic pPDLSCs **(I)** mediated nearly complete alveolar bone regeneration. **(K–Y):** HE staining showed new periodontal tissue regeneration in the periodontal defect area in autologous **(Q)** and allogeneic **(T)** pPDLSC groups. However, deep periodontal pockets and shortages of new bone and periodontal ligament fibers remained in the control **(K)** and HA/TCP groups **(N)**. The alveolar bone heights in the autologous **(Q)** and allogeneic **(T)** pPDLSC groups were much larger than those in the control group **(K)**, the HA/TCP group **(N)**, and PDLCs group **(W)**. Much thicker sulcular epithelia and enlarged epithelial pegs were evident in the control group **(L)**, the HA/TCP group **(O)**, and PDLCs group **(X)** compared with the autologous **(R)** and allogeneic **(U)** pPDLSC groups. Sharpy's fibers formed in the autologous **(S)** and allogeneic **(V)** pPDLSC groups, but typical Sharpy's fibers were not found in the control group **(M)**, the HA/TCP group **(P)**, or PDLCs group **(Y)**. **(Z):** (a) The percentage of periodontal bone in the autologous and allogeneic pPDLSC groups was significantly higher than those of the control group, HA/TCP group, and PDLCs group. (b) No positive staining of Y chromosomes was detected in the autologous pPDLSC group. (c) Positive staining of Y chromosomes was found in alveolar bone close to the PDL in the allogeneic pPDLSC group, indicating that regenerated periodontal tissues differentiated from allogeneic pPDLSCs. “D” indicates dentin, “C” indicates cementum, and “B” indicates bone **(K–W)**. **(A, F, K, L, M):** Control group without HA/TCP implantation, **(B, G, N, O, P):** HA/TCP group, **(C, H, Q, R, S):** autologous pPDLSC group, **(D, I, T, U, V):** allogeneic pPDLSC group, and **(E, J, W, X, Y):** autologous PDLCs group. The figures in the upper-right section of **(F–J)** are the coronal images of miniature pigs. Scale bar = 1 mm **(K, N, Q, T, W)**; Scale bar = 5 μm (**[Z]**, b, c). #, *p* < .01, compared with autologous PDLCs groups; *, *p* < .01, compared with autologous and allogeneic PDLSCs groups. Abbreviations: CEJ, cemeto-enamel junction; HA/TCP, hydroxyapatite/tricalcium phosphate; HAB, height of alveolar bone; PDL, periodontal ligament; PDLSC, periodontal ligament stem cell; SE, sulcular epithelium.

**Figure 3:**
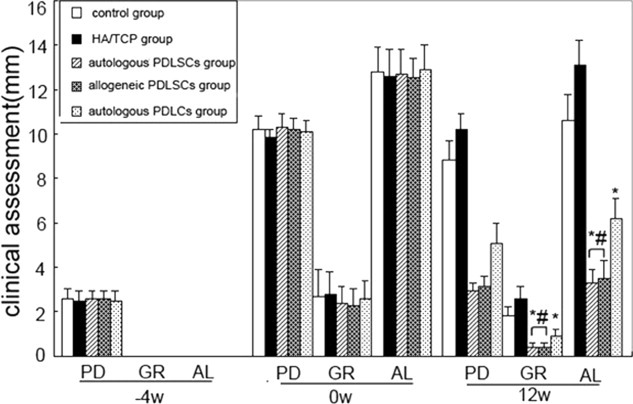
Clinical assessment of regeneration of periodontal tissues. At −4 week and 0 week, there was no significant difference among the five groups. However, 12 weeks post-transplantation, the PD, GR, and AL were significantly improved in autologous pig PDLSCs (pPDLSCs) or allogeneic pPDLSCs group than those in the control group, HA/TCP group, and autologous PDLCs group. *, *p* < .01, compared with autologous and allogeneic PDLSCs groups; #, *p* < .01, compared with autologous PDLCs groups. Abbreviations: AL, attachment loss; GR, gingival recession; HA/TCP, hydroxyapatite/tricalcium phosphate; PD, probing depth; PDLSC, periodontal ligament stem cell.

After generating minipig periodontitis model [9], allogeneic pPDLSCs sheet was transplanted into the periodontitis lesion areas (Supporting Information Fig. 1). At 12 weeks post-transplantation, the probing depth (PD) was 3.5 ± 0.6 mm in the allogeneic pPDLSCs group, 3.3 ± 0.4 mm in the autologous pPDLSCs group, 13.1 ± 1.1 mm in the HA/TCP group, 10.6 ± 1.3 mm in the control group, and 6.2 ± 0.7 mm in the autologous pPDLCs group. Statistical analysis indicated that both the autologous and allogeneic pPDLSCs treatments significantly improved periodontal tissue regeneration compared with the pPDLCs group, HA/TCP and control groups, with no difference between the autologous and allogeneic pPDLSCs groups (Figs. 2, 3).

**Figure 4:**
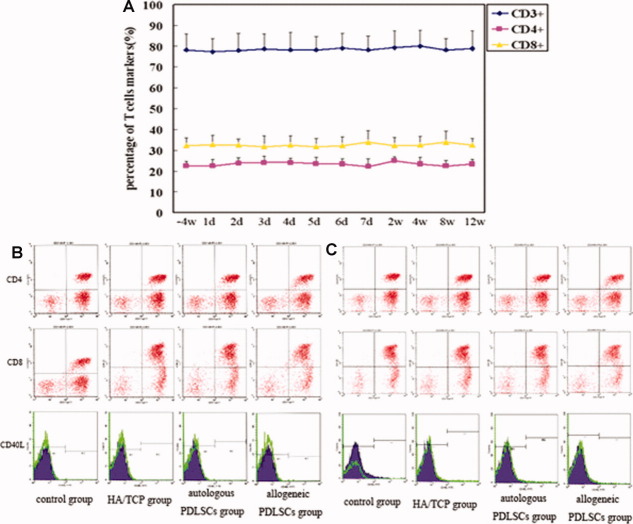
Dynamic evaluation of immunological parameters. **(A):** There were no significant differences in the indicated time points regarding the percentage of CD3^+^, CD4^+^, and CD8^+^ T cells in the allogeneic pig PDLSC transplantation group. **(B):** The expression of CD4^+^ and CD8^+^ T cells, as well as CD40L, a marker of activated T cells, were nearly identical among the four test groups 3 days post-transplantation. **(C):** The percentage of CD4^+^ and CD8^+^ T cells and the expression of CD40L were also nearly identical among the four groups 12 weeks after transplantation. Abbreviations: HA/TCP, hydroxyapatite/tricalcium phosphate; PDLSC, periodontal ligament stem cell.

Intraoral photographs indicated that 12 weeks after transplantation, pPDLSC-mediated periodontal tissue regeneration was close to normal level (Fig. 2C, 2D). Only limited or partial periodontal tissue was regenerated in the control group (Fig. 2A), HA/TCP group (Fig. 2B), and pPDLCs group (Fig. 2E). CT scanning showed that the height of the alveolar bone in the autologous and allogeneic pPDLSCs groups was recovered to almost normal levels. There was some new bone regeneration in the pPDLCs group, which was fewer than in the autologous and allogeneic pPDLSCs groups. However, the HA/TCP group and the control group showed little alveolar bone recovery (Fig. 2F–2J). In addition, histopathological photomicrographs indicated that new bone, cementum, and periodontal ligament were regenerated to normal levels in both the autologous and allogeneic pPDLSCs groups. In contrast, deep periodontal pockets along with bone and periodontal ligament defects were observed in the HA/TCP and control groups (Fig. 2K–2Y). These data indicate that a cell sheet from 2 × 10^6^ pPDLSCs could successfully regenerate periodontal bone defects of 3 mm × 5 mm × 7 mm in size (a sheet of 10^6^ pPDLSCs for an approximately 50 mm^3^ periodontal bone defect). The percentages of periodontium bone in the autologous and allogeneic pPDLSCs groups were 38.6% ± 2.3% and 35.7% ± 2.8%, respectively, which were significantly higher than those in the pPDLCs group (18.9% ± 3.3%), the control (10.1% ± 2.2%), and HA/TCP (8.3% ± 1.9%) groups (Fig. 2Z, a). Immunofluorescent assays revealed positive staining of Y chromosomes in the allogeneic pPDLSCs transplantation group (Fig. 2Z, c), compared with negative Y chromosome staining in the autologous pPDLSCs transplantation group (Fig. 2Z, b). To test the immunological reaction induced by allogeneic pPDLSC transplantation, we analyzed T cell-related immunological markers (Fig. 4), routine blood and biochemical tests, and immunoglobulin tests (data not shown) in whole blood. There were no significant differences between allogeneic and autologous PDLSCs groups based on the analysis of the samples, suggesting there were no immunological rejections in the animals that received transplantation of allogeneic pPDLSCs.

### PDLSCs Inhibit T Cell Proliferation Through PGE2

**Figure 5:**
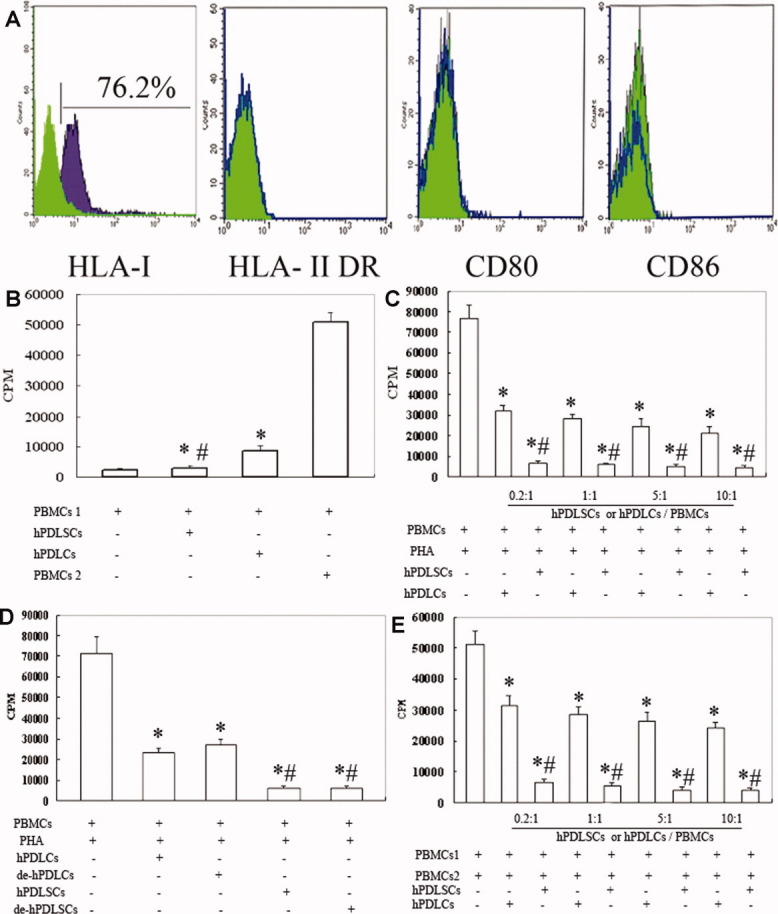
PDLSCs inhibit T-cell proliferation. **(A):** hPDLSCs were stained for immunological markers and analyzed by flow cytometry, revealing positive expression of HLA-I but negative expression of HLA-II DR, CD80, and CD86. Data represent five independent experiments. **(B):** Coculture of T cells with hPDLSCs showed that hPDLSCs failed to stimulate allogeneic T-cell proliferation. *, *p* < .01, compared with group of coculture of two allogeneic PBMCs; #, *p* < .01, compared with PDLCs groups. **(C):** T-cell proliferation stimulated by PHA in the presence or absence of hPDLSCs; the resulting inhibition was dose-dependent. *, *p* < .01, compared with PHA-stimulated PBMCs group; #, *p* < .01, compared with PDLCs groups. **(D):** T-cell proliferation induced by PHA was inhibited by delayed addition of hPDLSCs. *, *p* < .01, compared with PHA-stimulated PBMCs group; #, *p* < .01, compared with PDLCs groups. **(E):** A two-way mixed lymphocyte reaction (MLR) was inhibited by hPDLSCs in a dose-dependent manner.*: *p* < .01, compared with MLR group; #, *p* < .01, compared with PDLCs groups. Data are presented as mean ± SD of triplicates of six independent experiments. Abbreviations: CPM, counts per minute; HLA, human leukocyte antigen; hPDLC, heterogenic periodontal ligament cell; hPDLSC, human periodontal ligament stem cell; PBMC, peripheral blood mononuclear cell; PHA, phytohemagglutinin.

In vitro study revealed that hPDLSCs (76.2% ± 5.8%, *n* = 5) expressed human leukocyte antigen (HLA)-I, but were negative for immune phenotype markers HLA-II DR, CD80, and CD86 (Fig. 5A). To investigate the function of hPDLSCs as antigen-presenting cells in T-cell proliferation, preirradiated (20 Gy) hPDLSCs were cocultured with allogeneic normal PBMCs at a ratio of 1:1. These preirradiated hPDLSCs failed to stimulate allogeneic T-cell proliferation, whereas the allogeneic PBMCs triggered vigorous T-cell proliferation in the responder PBMCs. Moreover, hPDLCs could elicit low-grade T-cell proliferation, but significantly lower than allogeneic PBMCs (Fig. 5B). These results indicated that hPDLSCs displayed low immunogenicity. To determine whether hPDLSCs affect normal T-cell proliferation triggered by mitogen and alloantigen, various numbers of hPDLSCs (1.0 × 10^4^, 5.0 × 10^4^, 2.5 × 10^5^, or 5.0 × 10^5^) were cocultured with autologous PBMCs (5.0 × 10^4^) stimulated by PHA (0.5 μg/ml). PHA-induced T-cell proliferation was significantly inhibited by hPDLSCs in a dose-dependent manner, but identical numbers of autologous PBMCs showed no inhibitory effects, indicating that immunosuppression of T-cell proliferation was specifically attributable to hPDLSCs (Fig. 5C). Strikingly, hPDLSCs are capable of significantly suppressing T-cell proliferation when added into the culture dishes 2 days post-PHA stimulation (Fig. 5D). When two-way MLR was performed by coculture of PBMCs derived from two individuals and hPDLSCs collected from a third party, a cell number-dependent hPDLSCs inhibitory effect was found on PBMCs subjected to allogeneic PBMCs stimulation (Fig. 5E). In addition, whether added at day 0 or day 2, hPDLCs could also suppress the PHA-induced T-cell proliferation and two-way MLR, however, significantly lower than hPDLSCs. Taken together, these results indicate that hPDLSCs suppress allogeneic T-cell receptor-triggered or mitogen-stimulated T-cell proliferation in a cell number-dependent and antigen nonspecific manner, and their capability is higher than hPDLCs.

### PGE2 Plays a Crucial Role in PDLSCs-Mediated T-Cell Suppression

**Figure 6:**
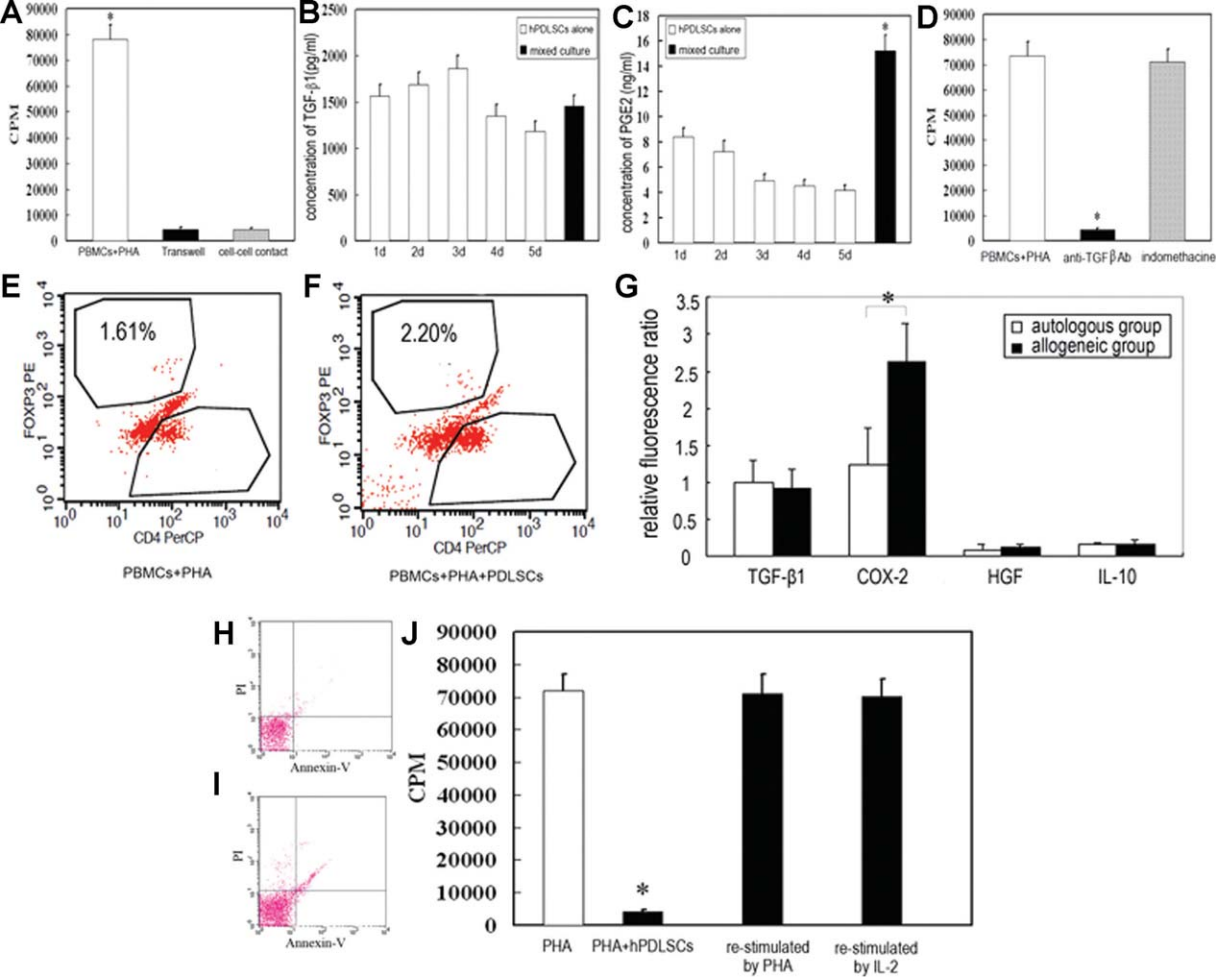
Prostaglandin E2 (PGE2) is the key inhibitor of T-cell proliferation mediated by PDLSCs. **(A):** T-cell proliferation was significantly inhibited when cocultured with human PDLSCs (hPDLSCs) in the Transwell culture system and in cell-cell contact culture. **(B):** TGF-β1 levels were similar between hPDLSCs alone and the coculture of hPDLSCs and PHA-induced T-cells. **(C):** The concentration of PGE2 significantly increased in the coculture of hPDLSCs and PHA-induced T cells. **(D):** The neutralizing monoclonal antibody against TGF-β failed to restore T-cell proliferation, while the PGE2 inhibitor indomethacine restored T-cell proliferation. **(E, F):** The percentage of Foxp3^+^ T cells in coculture of PBMCs and PHA **(E)** was similar to that in coculture of PBMCs, PHA, and hPDLSCs **(F)**. **(G):** The expression of COX2 was significantly higher in regenerated periodontal tissues of the allogeneic PDLSCs group than in the autologous PDLSCs group 2 weeks after transplantation, whereas TGF-β1, HGF, and IL-10 levels did not change (*n* = 4). **(H, I):** T-cell apoptosis rates were similar between PHA-stimulated T cells (**I**) and a coculture group with hPDLSCs **(H)**. **(J):** Inhibition of T cell proliferation mediated by hPDLSCs was restored when T cells were restimulated by PHA or IL-2. Data are presented as mean ± SD of triplicates of six independent experiments. *, *p* < .01. Abbreviations: CPM, counts per minute; HGF, hepatocyte growth factor; hPDLSC, human periodontal ligament stem cell; PBMC, peripheral blood mononuclear cell; PDLSC, periodontal ligament stem cell; PHA, phytohemagglutinin; TGF-β1, transforming growth factor β1.

We next explored mechanisms by which hPDLSCs suppress T-cell proliferation. To examine whether cell-cell contact was required for inhibition, a Transwell culture system was used to separate PHA-treated PBMCs from hPDLSCs. We found that T-cell proliferation was equally inhibited by cell-cell contact culture and the Transwell culture, suggesting that inhibition was independent of cell-cell contact and might involve in soluble factor(s) (Fig. 6A). As IL-10, HGF, TGF-β1, and PGE2 play important roles in bone marrow mesenchymal stem cell (BMMSC)-mediated T-cell immunosuppression [17–20], we used ELISA to quantify these four soluble factors in the culture supernatants of hPDLSCs and hPDLSCs cocultured with PHA-treated PBMCs. HGF and IL-10 were not detected in the supernatant of either hPDLSCs alone or the coculture. Kinetic studies revealed that the concentrations of TGF-β1 and PGE2 in the culture of hPDLSCs alone (1–5 days after seeding) were relatively stable (Fig. 6B, 6C). However, the supernatants from the coculture group contained PGE2 that were significantly elevated compared with those in hPDLSC group (Fig. 6C). The concentration of TGF-β1 was not significantly altered compared with the control group (Fig. 6B).

To pinpoint the identity of the factor(s) involved in immunosuppression, specific monoclonal antibodies against TGF-β, IL-10, and HGF, or the PGE2 inhibitor indomethacine, were added into coculture containing hPDLSCs, PBMCs, and PHA, and the restoration of T-cell proliferation was assessed. Only indomethacine restored the T-cell proliferation inhibited by hPDLSCs; the neutralizing monoclonal antibodies against TGF-β, IL-10, and HGF failed to restore T-cell proliferation (Fig. 6D). The percentage of Foxp3+ T cells in coculture of PBMCs and PHA (Fig. 6E) was similar to that in coculture of PBMCs, PHA, and hPDLSCs (Fig. 6F), indicating that TGF-β-induced active immunoregulation of regulatory T cells did not involve this immunosuppression. In addition to these in vitro data, real-time PCR assays showed that the expression of COX-2, a major enzyme in the biosynthesis of PGE2, was significantly increased in regenerated periodontal tissues of the allogeneic pPDLSC transplantation group, while the expressions of TGF-β1, HGF, and IL-10 were unchanged at 2 weeks post-transplantation (Fig. 6G). Taken together, our data suggest that PGE2 is a key inhibitor of T-cell proliferation mediated by PDLSCs. Moreover, we observed no difference in the number of apoptotic T cells between the coculture group containing PBMCs, PHA, and hPDLSCs, and the PHA-induced T-cell proliferation group; the majority of the T cells were viable, excluding T cell death as the major cause for the suppression (Fig. 6H, 6I), as previously reported [21]. In addition, we isolated T cells presuppressed by hPDLSCs for 5 days and restimulated with either PHA or IL-2 for another 2 days. We found that restimulation of T cells pretreated with hPDLSCs resulted in vigorous proliferation (restored to normal levels, Fig. 6J). Thus, hPDLSC-induced inhibition of T-cell proliferation is reversible in an anergic manner.

## DISCUSSION

Given their capacity to modulate immune responses, BMMSCs have been implicated as a potentially feasible treatment approach for several diseases, such as graft-versus-host disease [22] and autoimmune diseases [23,24]. The wide usefulness of BMMSCs can be attributed to their low immunogenicity and immunomodulation function. Recently, it was recognized that PDLSCs are a potential therapeutic cell source for tooth regeneration and reconstruction of periodontal ligament damaged by periodontal diseases [6,9,10]. The purpose of investigating immunogenicity and immunomodulation property of PDLSCs is to explore the allogeneic usefulness of PDLSCs in clinical application.

Initially, we observed similar biological and immunological properties in hPDLSCs and pPDLSCs (data not shown) [9]. Because of availability of antibodies to hPDLSCs and clinical application potential, hPDLSCs were selected for immunological analysis in vitro in this study. We have shown that hPDLSCs do not express HLA-II DR or costimulatory molecules and fail to elicit T-cell proliferation. This may contribute to the fact that hPDLSCs display low immunogenicity. To test the immunomodulation functions of hPDLSCs, we investigated the effects of hPDLSCs on PHA-induced allogeneic T-cell proliferation and on two-way MLR. hPDLSCs inhibit T cells in a dose-dependent manner both in a mitogen proliferative assay and in two-way MLR, indicating that PDLSCs inhibit the T-cell proliferation stimulated by mismatched major histocompatibility complex molecules. Moreover, T-cell proliferation induced by PHA was also suppressed by delayed addition of hPDLSCs, suggesting that hPDLSCs immunosuppress activated T cells analogous to dental root apical papilla stem cells [25]. In addition, although weaker than hPDLSCs, hPDLCs also possessed immunomodulatory effect, which were correspondent with the data on human synovial fibroblasts, dermal fibroblasts, lung-derived fibroblast, etc [26,27].

In previous studies, BMMSC-mediated immune suppression of activated T cells has been attributed to the secretion of antiproliferative soluble factors, such as IL-10, HGF, TGF-β1, and PGE2, even though contradictory results render unclear data regarding which factors play major roles in the process [17–20]. T cell-mediated immunity is considered to be one of the critical factors in pathogenesis of periodontitis [28]. We have demonstrated that hPDLSCs suppress T-cell proliferation via PGE2 in vitro. In addition, real-time PCR assays showed that the expression of COX-2, a major enzyme in the biosynthesis of PGE2, was significantly increased in regenerated periodontal tissues of the allogeneic pPDLSC transplantation group, while the expressions of TGF-β1, HGF, and IL-10 were unchanged at 2 weeks post-transplantation. Taken together, PGE2 is considered to play a crucial role in PDLSC-mediated immunomodulation and periodontal tissue regeneration.

The oral maxillofacial region of the minipig is similar to that of humans in anatomy, development, physiology, pathophysiology, and disease occurrence [29]. Thus, the minipig is a useful large animal model for dental orofacial research, including immunological studies. Here, we generated periodontitis lesions in the minipig, then tested the feasibility of using an allogeneic PDLSC sheet to repair the periodontitis-induced bone defects. As the typical immune response occur within 3 months post-transplantation based on previous studies of stem cell-induced immune responses [30,31], we examined tissue regeneration and immune reaction for a period of 12 weeks following transplantation. We found that the allogeneic PDLSCs contributed to periodontal tissue repair in a manner similar to the transplantation of autologous PDLSCs. Additionally, we observed neither rejections nor other damage in the allogeneic transplantation animals. However, the local immunological changes in the animals require further study.

We found that the periodontal tissue regeneration in control group without HA/TCP implantation was better than that in HA/TCP control group. The reason responsible for this phenomenon is not clear, may be associated with the recipient immune response to HA/TCP. However, the exact causes involved need further investigation.

Mesenchymal stem cells from other sources have been used to enhance periodontal tissue regeneration [32–34]. Here, we isolated PDLSCs from extracted third molars, which are usually regarded as a medical waste. Donor site morbidity limits the amount of bone marrow available for treatment, thereby extending the culture time is required to generate a large quantity for therapeutic use. PDLSCs harvested from periodontal ligament show high proliferation rate when compared with BMMSC and can differentiate into bone and soft periodontal tissues in vivo. It is required to conduct a well-designed study to compare the immunosuppressive and regenerative effects of PDLSCs with other MSC type in the future.

## CONCLUSION

Our study demonstrated that a sheet of minipig PDLSCs can repair allogeneic bone defects in an experimental model of periodontitis without detected immunological rejections, likely due to the low immunogenicity and immunosuppressive function possessed by PDLSCs. We developed a standard technological procedure for the application of allogeneic PDLSCs that could be effective in the treatment of periodontitis-induced bone defects.

## DISCLOSURE OF POTENTIAL CONFLICTS OF INTEREST

The authors indicate no potential conflicts of interest.
